# Beware the impact factor

**DOI:** 10.1007/s13280-016-0777-6

**Published:** 2016-03-28

**Authors:** Kevin J. Noone

**Affiliations:** Environmental Science and Analytical Chemistry (ACES), Stockholm University, 106 91 Stockholm, Sweden

The research community is under increasing pressure to document the impact of its activities. One of the root causes of this pressure is the entirely reasonable desire on the part of the funders of research—and ultimately society at large—to know what benefits they are accruing on their investments. Done judiciously, an ongoing assessment of the impact of research would be a good thing for all involved: society, funding agencies, and the research community itself. Done injudiciously, such assessments can be powerfully counterproductive. Judging impact begs the obvious but often ignored question: impact on whom or on what?

Assessments require metrics on which to draw conclusions about impact. In the research community, the *h* factor (Hirsch 2005) is usually used to assess the impact of an individual’s research efforts and has been extended to institutions (Prathap 2006) and journals (Moussa and Touzani 2010), while the *impact factor* has become another metric for judging the quality of journals in which research results are published (Garfield 2006). One recent example of the pressure to use these metrics comes from the governing board of a Norwegian development and aid research program. The Norway-Global Partner (NORGLOBAL) program is sponsored by the Norwegian Research Council and the Norwegian Agency for Development Cooperation (Norad). It is very broadly interdisciplinary in nature, and as such presents special challenges in terms of impact assessment. At a recent meeting of the governing board of the NORGLOBAL program (Oslo, 26 January 2016), representatives from Norad expressed the desire to include the impact factor of the journals in which results from the research funded through the program are published as part of final reports for projects. On the surface, this would seem to be a reasonable request. On deeper consideration, this may be leading us down the wrong path.

Like the Gross Domestic Product (GDP) for measuring economic impact, these metrics have become defaults in terms of assessing the quality and impact of research efforts. In the 1934 report that defined the concept of GDP (Kuznets 1934), Simon Kuznets and co-authors wrote “*A student of social affairs who is interested in the total productivity of the nation, including those efforts which, like housewives services, do not appear on the market, can therefore use our measures only with some qualifications.*” Similarly, I suggest that *h* and *impactfactors* should only be used to assess research impact “with some qualifications”. This criticism is not new; the utility of the *h* factor has been widely discussed in the literature (Lehmann et al. 2006; Bornmann and Daniel 2008). Criticism of these indices includes the difficulty of comparisons across disciplines, comparing academics at different stages in their careers, and the placement of individuals in the author list. Using the *h* or *impact factors* is particularly problematic in areas such as development research, since they are dominated by journals (and the associated logic, cognitive values and perspectives) from the global North published in English.

I can provide one example of the limitations of these metrics for determining impact from personal experience. It was a competition for my time between two activities; one activity was helping a PhD student write a series of papers on a new analytical technique for determining the sources of biogenic aerosol particles. The work involved very detailed analytical chemistry, and was focused on a specific issue that has been confounding atmospheric chemists. One of the papers (Gonzalez et al. 2014) was selected as a “hot” article by the journal *Environmental Science: Processes and Impacts* (which, since we are on the topic, has an impact factor of 2.171). This paper represented sterling scientific work on the part of the then PhD student, as evidenced by the “hot” characterization. The other activity was working with a panel convened by the private-sector firm DNV GL to help them develop a strategy for their sustainability efforts. DNV GL has roughly 16 000 employees in more than 100 countries. One of the reports from this effort—“A Safe and Sustainable Future: Enabling the Transition” (Hultmann and Koefoed 2014)—has been used in both internal training activities for the company, and as support for DNV GL’s work with their own customers on sustainability issues.

Which of these publications had greater impact? According to the metrics we currently use, the answer is easy: the research paper in the specialist scientific journal. It will contribute to the *h* factors of all the co-authors, and if asked we can even cite the journal’s impact factor. Since it is still young as a publication, it is perhaps not surprising that the number of citations is low; it has been cited once in the two years since it was published (Jacobsen and Anthonsen 2015) in another very focused article in a specialized journal. We can be numerically precise in specifying impact factors in these cases, but I would maintain that this precision is not matched by an equal level of meaning. However, if we are to broaden our classification of impact, the answer may change. The DNV GL report has influenced how a major multinational company pursues its own sustainability efforts, and how it interacts with other companies to help them become more sustainable in their operations. Bjørn K. Haugland, Executive Vice President and Chief Sustainability Officer at DNV GL, writes the following about the report:

DNV GL’s vision is “Global Impact for a safe and sustainable future”. Hence, understanding the global sustainability agenda is essential for our strategic development. The development of the report was a collaborative process that harnessed input, and was challenged by thought leaders from around the world. The report and supporting material like interviews and videos made it possible for us to engage and guide our 16 000 employees and 80 000 customers on how we put our vision into action in all the 100 countries we operate in. The report focused in particular on sustainable development in our five industry sectors and we arranged seminars globally throughout 2014 in order to engage our stakeholders. 15 000 copies of the report were distributed together with extensive leverage through social media

From a societal benefit perspective, the impact of the DNV GL report is very much larger than the impact of the journal article. According to our metrics, however, the impact of the DNV GL report is zero. In fact, I can easily argue that working on this report resulted in a negative impact—in two ways. First, the time I spent working with DNV GL was not spent publishing specialized papers or writing proposals for research projects on arcane scientific issues—activities which sooner or later would have ticked the current boxes for impact. Second, in principle my University has societal interaction as one of its main tasks alongside research and teaching. In reality, activities like contributing to the DNV GL report are neither captured nor rewarded in our system. They are at best invisible, but more likely negative since again this was time not spent on research or teaching activities.

I have the luxury of being sufficiently senior that I need not care much about burnishing my *h* or *impact factors*. The situation for my colleagues earlier on in their careers is not so benign. The strong signals being provided to early-career researchers are to concentrate on activities that often isolate them from society at large. I feel this is a mistake. There has been a call for a broader set of metrics for impact—altmetrics (Priem et al. 2010), but so far such metrics have not achieved much traction. How do we improve the situation?

To a large extent, the research community itself is at fault for allowing ourselves to arrive at this point. We have not expended sufficient effort in developing metrics that more properly and completely reflect the utility and impact of the work we do. We need to become much smarter and more strategic in how we measure success in the research field. Developing better metrics is not simple. I have had the opportunity to participate for some time in discussions on this topic from several perspectives: as an individual scientist, as representative of a large international research organization (the International Geosphere-Biosphere Programme), as Swedish representative to the International Group of Funding Agencies (IGFA), and as a member of the Transdisciplinary Advisory Board for the Joint Programming Initiative on Climate (JPI Climate). A recent (28–29 September 2015) workshop in Brussels was sponsored by JPI Climate, involving both researchers and stakeholders—a group well qualified to come up with ideas for improved metrics. This workshop resulted in dozens of suggestions for success criteria for the program, but only three suggestions for metrics by which to judge them. There seems to be near universal recognition that the current metrics are inadequate, but little consensus on how to create better ones. There does not appear to be any sort of consensus among the research community and society at large as to what should be measured to assess impact.

Creating better metrics for impact will require assembling and convening an international group of experts with experience from many disciplines in the research domain. The group should include representatives from stakeholder communities for which research is important. It should also include representatives from the groups that are footing the bill for our research endeavors. The group should pay particular attention to metrics that adequately capture the value of inter- and trans-disciplinary work. It should not be afraid to include aspects of quality that are difficult to quantify numerically, but are essential for judging the true impact. It should be convened by an organization or organizations with sufficient authority that the results of the effort will be respected. The effort should be supported by financial and human resources at a level matching the dignity of the work to be done.

Fortunately, there are solutions to this conundrum. Organizations like the International Council for Science and the International Social Science Council have the convening authority and the organizational infrastructure to carry out a task of this kind. I would like to challenge these organizations to undertake this effort on behalf of the research communities they serve, and to the benefit of both science and society.
